# Design and validation of Segment - freely available software for cardiovascular image analysis

**DOI:** 10.1186/1471-2342-10-1

**Published:** 2010-01-11

**Authors:** Einar Heiberg, Jane Sjögren, Martin Ugander, Marcus Carlsson, Henrik Engblom, Håkan Arheden

**Affiliations:** 1 Department of Clinical Physiology, Lund University and Lund University Hospital, Lund, Sweden

## Abstract

**Background:**

Commercially available software for cardiovascular image analysis often has limited functionality and frequently lacks the careful validation that is required for clinical studies. We have already implemented a cardiovascular image analysis software package and released it as freeware for the research community. However, it was distributed as a stand-alone application and other researchers could not extend it by writing their own custom image analysis algorithms. We believe that the work required to make a clinically applicable prototype can be reduced by making the software extensible, so that researchers can develop their own modules or improvements. Such an initiative might then serve as a bridge between image analysis research and cardiovascular research. The aim of this article is therefore to present the design and validation of a cardiovascular image analysis software package (Segment) and to announce its release in a source code format.

**Results:**

Segment can be used for image analysis in magnetic resonance imaging (MRI), computed tomography (CT), single photon emission computed tomography (SPECT) and positron emission tomography (PET). Some of its main features include loading of DICOM images from all major scanner vendors, simultaneous display of multiple image stacks and plane intersections, automated segmentation of the left ventricle, quantification of MRI flow, tools for manual and general object segmentation, quantitative regional wall motion analysis, myocardial viability analysis and image fusion tools. Here we present an overview of the validation results and validation procedures for the functionality of the software. We describe a technique to ensure continued accuracy and validity of the software by implementing and using a test script that tests the functionality of the software and validates the output. The software has been made freely available for research purposes in a source code format on the project home page http://segment.heiberg.se.

**Conclusions:**

Segment is a well-validated comprehensive software package for cardiovascular image analysis. It is freely available for research purposes provided that relevant original research publications related to the software are cited.

## Background

Applied medical research is becoming more and more dependent on imaging for evaluation of the therapeutic effects of new drugs or therapies. Thus, dedicated image analysis software is needed for quantitative medical imaging. Commercially available software often offers limited functionality and frequently lacks the validation that is required for clinical studies. On the other hand, open source software offers transparency and the ability to modify the source code is well-suited to academic research since it gives researchers the ability to see exactly how the algorithms are implemented. The current trend among research grant organisations is that the results of government-funded projects should be published in open access journals or should be otherwise publicly available. Consequently, open access publishing has had a noticeable effect on the ease with which scientific results become available.

Publication of results in peer-reviewed journals is the traditional way of documenting and mediating progress in science. For classical sciences such as medicine and physics, this is normally sufficient since the reader can incorporate the information given in the publication into his or her own research. However, medical image analysis research often involves complex algorithms, and one cannot easily incorporate the results into one's own research since the algorithms described in the scientific papers usually need to be re-implemented for use by other research groups [1]. Thus, we believe that releasing medical image analysis software to other research groups as freeware has the potential to have a profound effect on medical image analysis and applied medical imaging research. If the source code is reusable and written in a standardized way, the application can be modified or extended with new algorithms. This would permit the development of new algorithms or refinement of existing algorithms in order to satisfy requirements that arise in a clinical research setting. Furthermore, scripting capabilities for medical image analysis software may open up new lines of research that were previously untestable, since manual analysis would not have been feasible. For example, in a recent study it was possible to classify 72 regional myocardial sectors according to the neighbouring sectors and to track them in 22 patients over 5 points in time, thus generating over 50,000 classified data points [2]. This task would have been impossible without advanced scripting capabilities in the analysis software.

The Segment cardiac image analysis software package, which is the subject of this article, was originally developed by the first author and was released in 2005 on a freely available basis. Since then it has been downloaded by more than 2,000 unique users in 74 countries, and approximately 300 research groups. To date, it has been referenced in more than 40 scientific publications. One advantage of freely available medical image analysis software is that it facilitates multi-centre clinical trials since all the participating sites can use the same software. The authors are aware of two ongoing multi-national and multi-centre studies that are using Segment software. Until recently, the software was distributed as a precompiled Windows application. If the software was available in a source code format, other researchers could contribute with their own modules and improvements. Image processing experts could then directly integrate new features into the software and make these improved algorithms available to other researchers. In this way, algorithm developers would be able to focus on algorithm development and make use of the common basic functionality of the software such as image loading, image display, user interactions etc. We believe that this may not only increase scientific productivity, but more importantly it may also provide a bridge between the very latest image processing ideas and applied clinical research. Today, many very promising image processing ideas never reach clinical routine because it is too cumbersome to write prototype software that is sufficiently user-friendly to be used by clinical researchers. Thus, the aim of this article is to present the design and validation of a cardiovascular image analysis software package and to announce its release in a source code format.

## Implementation

What follows is an outline of some of the important details concerning design and implementation of the software.

### A. Overview

**Figure 1:**
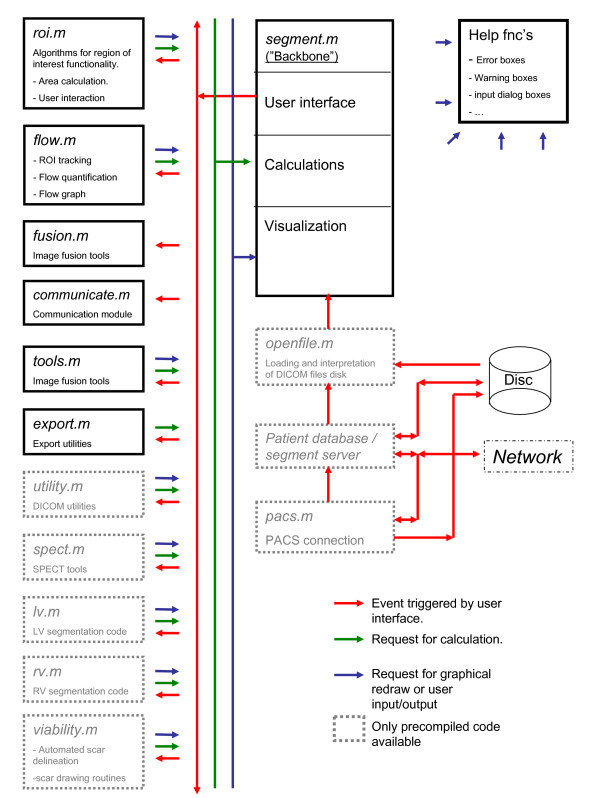
**Overview of the main building blocks of Segment and transaction analysis**. Red arrows indicate communication that is initiated from a user interface. Green arrows indicate call of calculation sub-routines. Blue arrows indicate requests for graphical update or call of low-level user input and output routines.

The software can be divided into 14 main functional blocks. An overview of how these building blocks communicate and relate to each other is given in Figure 1. Each block is implemented as a separate Matlab file and documented in detail in the technical manual. Closer descriptions of each building block are beyond the scope of this article. The grey blocks are not included in the source code, but will be accessible as precompiled code.

An overview of the image processing algorithms is given in Table 1, an overview of manual image processing tools is given in Table 2, and finally the export, import and reporting capabilities are described in Table 3. For each algorithm or functionality, appropriate references to publications where the functionality has previously been described, if applicable.

**Table 1:** Automated or semi-automated image processing tools in Segment

Algorithm	Dimensionality	Reference	Section
***Ventricle segmentation***			
- Left ventricle	2D, 2D+T, 3D, 3D+T	[5]	C.
- Semi-automatic tools for right ventricle	2D, 2D+T, 3D, 3D+T	*	C.

***Flow***			
- Phase unwrapping algorithm	2D+T, 3D+T, 3+3D+T	*	D.
- Phase background correction	2D+T, 3D+T, 3+3D+T	*	D.
- Automated vessel tracking	2D+T, 3D+T	*	D.
- Flow visualization	2D+T	*	D.

***Delayed enhancement/viability***			
- Quantification of infarct size	3D	[3,4]	H.
- Infarct extent	3D	[11,22]	H.

***General Object Segmentation***			
- Fast levelset	3D, 2D+T, 3D+T	[25]	I.
	3D, 2D+T, 3D+T	*	I.

***SPECT***			
- Left ventricle segmentation	3D	[28]	J.
- Defect size	3D	#	J.
- Gated SPECT segmentation	3D+T	#	J.

Dimensionality: 2D) works on two dimensional images, 2D+T) works on time resolved two dimensional images, 3D) works on three dimensional images, 3D+T) works on time resolved three dimensional images, 3+3D+T) works on three component three dimensional time resolved images. Reference: [X]) previously published in reference X, #) previously unpublished data, manuscript submitted, *) algorithm presented for the first time in this study. Section: Refers to the Result section where functionality is described.

**Table 2:** Manual image processing tools in Segment

Algorithm	Dimensionality	Ref	Section
***Image visualization tools***			
- Contrast adjust + auto contrast	2D, 2D+T, 3D, 3D+T, 3+3D+T	*	B.
- Multi view/panel support	2D, 2D+T, 3D, 3D+T, 3+3D+T	*	B.
- Image plane intersection	3D, 3D+T, 3+3D+T	*	B.

***Manual contouring tools***	2D, 2D+T, 3D, 3D+T	*	E.

***Region of interest analysis (ROI)***			
- Signal intensity quantification	2D, 2D+T, 3D+T	*	F.
- Histogram analysis	2D, 2D+T, 3D+T	*	F.
- Visual ROI analysis	2D, 2D+T	*	F.
- Area tools	2D,2D+T	*	F.
- Volume tools	3D,3D+T	*	F.

***Linear measurements***	2D, 2D+T	*	G.

***Annotation points***	2D, 2D+T, 3D, 3D+T	*	G.

***Image fusion***	3D	[27]	K.

***Reformating image tools***			
- Multi planar reconstruction	3D, 3D+T	*	L.
- Resampling	2D, 2D+T, 3D, 3D+T	*	L.

Dimensionality: 2D) works on two dimensional images, 2D+T) works on time resolved two dimensional images, 3D) works on three dimensional images, 3D+T) works on time resolved three dimensional images, 3+3D+T) works on three component three dimensional time resolved images. Reference: [X]) previously published in reference X, #) previously unpublished data, manuscript submitted, *) algorithm presented for the first time in this study. Section: Refers to the Result section where functionality is described.

**Table 3:** Export, import and reporting capabilities of Segment

Algorithm	Dimensionality	Reference	Section
***DICOM import and manipulation***	2D, 2D+T, 3D, 3D+T	*	A

***Movie recording capacity***	-	*	B

***Wall thickening analysis***	2D+T, 3D+T	*	C

***Polar plot of function and infarct***	3D, 3D+T	[29]	M

***Batch export to statistical software***	-	*	N

***Communication module to facilitate multicenter trials***	-	*	O

***Plug-in capabilities***	-	*	P

Dimensionality: 2D) works on two dimensional images, 2D+T) works on time resolved two dimensional images, 3D) works on three dimensional images, 3D+T) works on time resolved three dimensional images, 3+3D+T) works on three component three dimensional time resolved images, -) dimensionality not applicable. Reference: [X]) previously published in reference X, #) previously unpublished data, manuscript submitted, *) algorithm presented for the first time in this study. Section: Refers to the Result section where functionality is described.

Each loaded image or image stack (a single image (2D), a time-resolved single image (2D+T), a stack of a images covering a multi-slice image volume (3D) or a time-resolved multi-slice image volume (3D+T)) is stored as a struct, and has fields for storing contours, image data, image orientation, resolution, image acquisition details, delineations, annotations and measurements. Handles to the graphical user interface and some temporary data are stored in a global data structure. A set of low-level input and output user interface routines was implemented to improve portability of the code. An object-oriented system for graphical user interfaces is employed to improve the ability to maintain the code and simplify development of new user interfaces.

**Figure 2:**
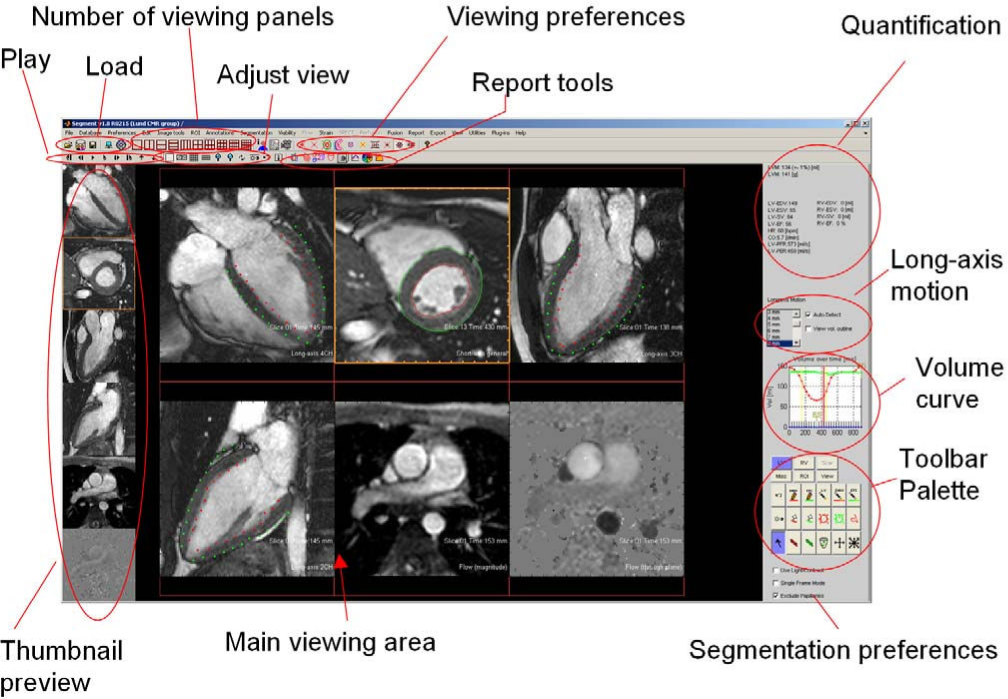
**Annotated screen shot of the main user interface of Segment**. The circles indicate functional units in the user interface. Example images from one patient have been loaded and displayed in different viewing panels. The yellow box around one image panel indicates the current image stack.

The software was designed with clinical research in mind, and to maximise work flow and user-friendliness. A screen shot of the main graphical user interface is shown in Figure 2. The internal file format used by Segment was designed so that a complete patient examination can be stored together with all measurements and annotations. This allows the clinical researcher to go back and see how the delineations were made, which is often not possible with commercially available software packages. The internal file format also allows the user to batch process multiple data sets and to export quantitative data in a spreadsheet format.

### B. Programming environment

The software package is written in Matlab and, for time-critical sections of the code, standard ANSI-C is used coded with Mex-wrappers so that they can be called from Matlab. The complete software project consists of about 90,000 lines of Matlab code and about 10,000 lines of C code. In total, there are 44 separate user interface panels. Proper version control software is necessary when managing a programming project of this size. Version control is managed using the open source solution Subversion (SVN, http://subversion.tigris.org) with Tortoise SVN http://tortoisesvn.tigris.org as a shell extension. Feature requests and bug reports are managed using the web-based open source software Trac http://trac.edgewall.org. Up until now, the whole application has been compiled as a stand-alone application and distributed together with the Matlab Compiler Runtime Environment. Upon publication of this article, the source code, the user manual and the technical manual will be available on the Segment home page http://segment.heiberg.se. Precompiled versions of Segment will still be available for Windows and Linux (Ubuntu distribution). Precompiled object files for the C code will be made available for 32-bit and 64-bit Windows operating systems, 32-bit and 64 bit Linux, and Mac OS X.

### C. Stability, accuracy and validity

For clinical image analysis, the following requirements of a software package are crucial: (1) stability, (2) high performance, and (3) accuracy and validity.

To achieve stability, the software was designed so that even when run-time errors occur, the application should not crash and the user interface should not end up in a state in which the user must to restart the application.

To optimise performance, effective memory management and highly optimised routines are essential when designing the software. Cardiac imaging is particularly demanding since the data sets are frequently large and multidimensional with regard to space and time. Great care was taken to avoid duplicating image data unless absolutely necessary. Also, time-critical routines were optimised and coded in C.

To achieve accuracy and validity, validation considerations were incorporated into the design process by placing all calculations of distances, areas, volumes and region of interest mask generation in well-validated sub-functions. Care was taken at the design phase to avoid loss of accuracy due to loss of numerical precision. One such consideration was to represent contours, measurements and regions of interest with double-precision floating point numbers. Surprisingly, this is not often the case in commercially available software tools where pixel-based approaches are frequently used, which can be quite misleading--especially in small regions of interest. Even with a robust underlying design, it remains a challenge to maintain a strict quality policy that allows use for clinical research. The solution that we have chosen is to write an extensive test script that runs on archived test data. The test script output from the software is then compared with known accurate results from previously validated scientific publications. To ensure that the software maintains a high standard, the complete software repository will only be made available to a limited number of trained developers and incorporation of user-contributed code in the code base will only be done after careful testing and quality control. With the use of the test script, it is possible to quickly test the entire software project and uncover unexpected side effects when the code is modified. Coding standards and quality policies are given in the technical manual, which is available on the project home page.

### D. Software maintenance

Medical imaging is developing rapidly and as a result medical image analysis software must be continuously refined and maintained. To ensure long-term maintenance of the code, we have chosen a solution whereby a company was formed to support and commercialise the software for use in clinical practice or by commercial users. Lund University is a shareholder in this company, and researchers have and will continue to have access to the current and future versions of the source code of the software.

### E. Terms of licence

Segment is freely available for academic investigational research use (studies paid by government-derived funds or donations) provided that the original research publications relevant to the software are cited. The software is also free for educational purposes. Note that the license terms do not generally include trials paid by pharmaceutical companies. For commercial use, Segment is sold and supported by the company Medviso AB, Lund, Sweden. Individuals or organisations are not allowed to compile software products derived from Segment that are to be sold commercially or shipped together with other commercial products without written permission from Medviso AB.

## Results

Segment is a full-featured software tool for cardiovascular image analysis and to date, it has been used in a wide range of publications ranging from technical algorithm descriptions [3-5] to applied research on the effects of cardiac gene therapy [6], perfusion MRI [7], perfusion multidetector CT [8], applied human physiology [9], validation of an imaging technique in clinical cardiology [10], analysis of infarction with MRI [11] and MDCT [12], and analysis of microinfarction [7], regional cardiac function [13], for the first time quantitatively determine the infarct evolution in man [14], brain imaging [15] and also experimental imaging in rodents [16]. The software has been developed with a view to its use in cardiovascular magnetic resonance imaging (MRI) and myocardial perfusion single-photon emission computed tomography (SPECT), but in principle it can be used for image analysis in any organ system, and it has also been used for image analysis in computed tomography (CT) and positron emission tomography (PET). The following sections deal with the main features of Segment. Each section presents an overview of validation results and procedures where applicable.

### A. Loading of DICOM images

DICOM images from all major MRI vendors, including both human and animal scanners (Bruker, GE, Philips, Siemens and Varian) can be loaded into the software. Correctness of the loaded image data is validated by the test script, which loads a large number of image types from all vendors and compares the results to previously manually validated results using ImageJ or proprietary vendor software. Checks are made for correct image sorting, image resolution, time increment between time frames, slice thickness etc.

### B. Image display

A large set of image display tools has been implemented. Examples of functionality include simultaneous display of multiple image stacks, viewing of contours and regions of interest, image stack intersections, adjustment of image contrast and brightness, and scrolling over time and between image slices. Each image stack can have its own colour scale or contrast settings, and 12-bit color mapping is used internally. The user interface was developed to maximise the image display area without compromising user friendliness. Furthermore, multiple monitors are supported to maximise the display area and improve the work flow. Image display tools cannot be evaluated quantitatively, but the functionality is carefully tested by the test script. Tools for recording movies are also incorporated in the software. This greatly facilitates preparation of scientific presentations that include movies.

### C. Automated segmentation of cardiac ventricular dimensions in MRI

Automated segmentation of the left ventricle in MRI was the first image process algorithm implemented in Segment. The algorithm has been described and validated [5,17]. There was an excellent correlation between automated segmentation and manual segmentation for end diastolic volume (EDV), R^2 ^= 0.99, with a mean error of -1 ± 11 ml, and left ventricle mass (LVM), R^2 ^= 0.94, with a mean error of 4 ± 15 ml [5]. Tools for semi-automatic delineation of the right ventricle have also been incorporated. From the automatically or semi-automatically segmented surfaces, wall thickness, wall thickening, and fractional wall thickening can be calculated [5,17]. Typical computational time for a standard Windows XP desktop PC (Intel Dual Core 2 GHz, Buss speed 770 MHz and 2 GB RAM) is about 10 seconds for a typical data set with 12 slices, and 30 timeframes.

### D. Flow quantification

Flow measurements are of major importance in cardiovascular research, and velocity encoded phase contrast (PC) magnetic resonance imaging (MRI) is the golden standard for the *in vivo *quantification of blood flow in large vessels. It has been shown that modern MRI scanners may have phase offsets due to eddy currents, which can have a large effect on clinical flow measurements [18]. Segment has tools for compensation of linear and higher-order background phase offsets due to eddy currents or Maxwell effects [19]. It is possible to use automatic detection of stationary tissue based on the temporal standard deviation of the phase or to use manual regions of interest.

When imaging velocities higher than the chosen velocity encoding range, phase aliasing will occur and cause wrap-around artifacts in the quantitative visualization of velocities. Tools to compensate for such aliasing effects have been implemented and used successfully [20]. The algorithm detects temporal discontinuities in the phase, and pixels with a temporal phase jump pair are unwrapped. The tool also includes a graphical user interface for manual correction.

**Figure 3:**
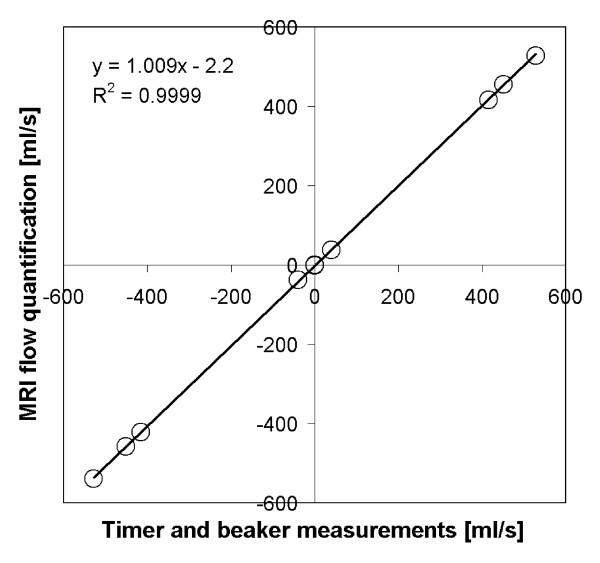
**Correlation plot where timer and beaker flow measurements are plotted versus velocity encoded MR flow quantification**.

**Figure 4:**
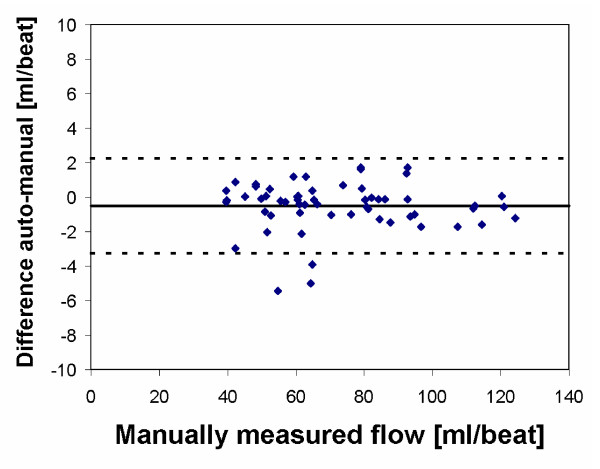
**Difference in total net flow comparing automated and manual vessel delineation**. Bias ± 2 SD is indicated in the plot.

**Figure 5:**
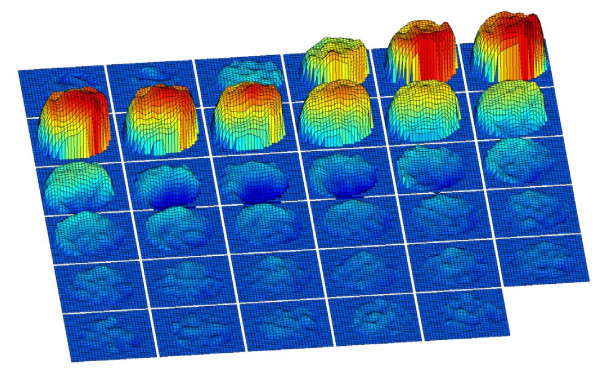
**Example of vessel flow profile visualisation over time in the human aorta of a healthy volunteer**. The first time frame is at the top left and time is increasing along each row. Top right vessel is peak systolic time frame. Note the relative skewedness of the flow in the healthy volunteer.

Validation of flow measurements has been performed both in phantom experiments and in patients. Phantom measurements were performed using gravity-driven flow at 5 different flow rates through a silicon gel with two holes 26 mm in diameter. The true rate of flow was measured by beaker and timer. The agreement between measured flow and beaker and timer was excellent (y = 1.009 x - 2.2 ml, R^2 ^= 1.00). Figure 3 shows a correlation plot for this experiment. In patient images, one experienced observer outlined the ascending and descending aorta in 32 patients. In total, 64 regions of interest were analysed both manually and using automated vessel delineation. In the automated vessel delineation, the manually outlined vessel contour from the first time frame was taken as input to the algorithm. In 4 of the 64 vessels (6%), the automated vessel tracking failed due to poor image contrast and imaging artifacts, and resulted in a difference greater than 10 ml and large visual over-estimation in vessel area. These vessels were excluded from further analysis. Bias and variability between total net flow for the manual vessel delineation and automated vessel delineation was -0.5 ± 2.8 ml/beat for the remaining 60 vessels. Figure 4 illustrates the difference in total net flow between automated and manual vessel delineation. Total time for automated vessel tracking is about 1.5 seconds on an ordinary desktop PC for a typical data set with 35 time frames. Besides the quantitative tools for flow mapping, there are also visualisation tools for visualisation of flow profiles. Figure 5 illustrates vessel flow profiles over time. The first time frame is at the top left, and time is increasing along each row.

### E. Tools for drawing object contours

All the necessary tools for manually drawing object contours, and regions of interest, linear measurements and annotations are implemented. The same tools can be used transparently to correct automated delineated object contours. All drawing tools include full undo capabilities. All quantitative measures from object contours rely on the same low-level quantification algorithms. These low-level algorithms were validated using computer phantoms implemented in the software, and are included in the test script.

### F. Region of interest analysis

Tools for analysing regions of interest (ROIs) are implemented. Details about signal intensities and area are available. Areas of the regions of interest are measured by accurate polygon calculations, but signal intensity and signal intensity statistics are calculated by performing statistics on discrete pixels inside the ROI. Basic statistics such as mean intensity and standard deviations over time are available. Typical calculation time for calculating statistics for one ROI in a typical data set with 35 time frames is about 0.2 s on an ordinary Windows XP desktop PC, Intel Dual Core 2 GHz, Buss speed 770 MHz and 2 GB RAM.

Image intensities are stored in Segment as single precision floating-point numbers and internally scaled into the range 0-1. Signal intensity measurements are done with single-precision arithmetics which give relative errors of about 10^-6^). However, note that the DICOM standard specifies storage of images using between 12 and 16 bits. For CT images, they are internally stored in Segment as 16-bit signed integers and are converted to single-precision floats before any arithmetics besides general object segmentation or display.

**Figure 6:**
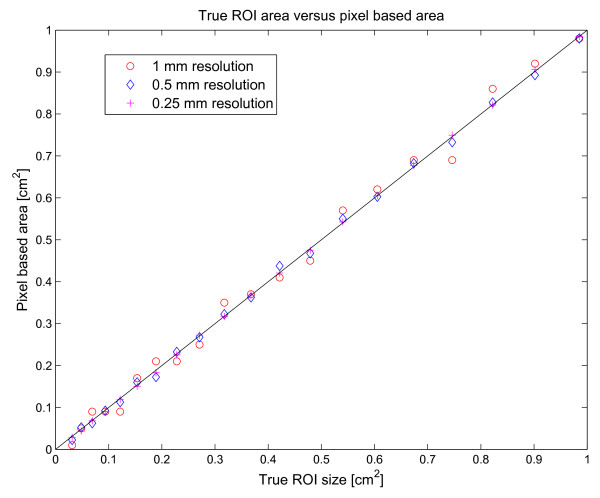
**Illustration of sampling error for small regions of interest**. Horizontal axis represents true area and vertical axis represents area based on counting pixels included in the ROI. The error depends on the pixel resolution. Open circles denote a pixel resolution of 1 mm, diamonds indicate a pixel resolution up-sampled to 0.5 mm, and plus signs indicate a pixel resolution up-sampled to 0.25 mm.

Detailed statistics such as distribution are available as histograms of pixel intensities. Measuring signal intensities in small ROIs using discrete pixels introduces a sampling error. This sampling error is quantified in Figure 6 for circular ROIs. For regions of interest of a size between 0 and 1 cm^2^, the corresponding error is 0.07 ± 2.5 for a pixel resolution of 1 mm, -0.17 ± 0.88 for a pixel resolution of 0.5 mm, and -0.1 ± 0.27 for a pixel resolution of 0.25 mm. Measurements for maximum values, minimum values, and full-width half maximum values, and minimum and maximum temporal derivatives can also be exported. It is also possible to apply smoothing prior to these calculations. The optional smoothing applied is a user-adjustable Gaussian filtering, and is implemented using Normalized Averaging to account for edge effects at the beginning and end of the signal.

### G. Linear measurements and annotation points

Linear measurements can be made in single time frame and the distances between start and end points are calculated as the standard Euclidian distance. Distances and positions are stored as double-precision floats, and thus positions and distances can be measured with sub-pixel accuracy. Annotation points, both static in time and time-resolved, can be placed in the image volume, and coordinates for these annotation points can be exported. This also allows measurement of distances in time-resolved three-dimensional space.

### H. MRI viability analysis

Delayed contrast enhancement MRI can be used to differentiate between viable and necrotic or fibrotic tissue, owing to changes in the extracellular distribution volume after irreversible cell injury [21]. Automated tools for delineation of hyper-enhanced regions are implemented and have been clinically validated [3] and used in several studies, such as [22] and [11]. Hyper-enhanced regions can be expressed in ml, as a percentage of left ventricle mass, or as endocardial extent [22]. Furthermore, the method for delineation of hyper-enhanced regions has been improved with a novel paradigm to account for the partial volume effect, where the hyper-enhanced pixels are weighted depending on the image intensity [4]. The method was validated in 8 animals by *in vivo *MRI compared to high-resolution *ex vivo *MRI. Further validation was performed in 40 computer phantoms and 40 patients, by comparing the results to manual delineations from three experienced observers. Values of mean bias ± variability (or standard deviation), expressed as a percentage of left ventricular myocardium (%LVM), were -0.3 ± 1.3% (animals), -1.2 ± 1.7% (phantoms) and 0.3 ± 2.7% (patients). The new weighted algorithm had lower variability than the previously published approach of dichotomously classifying pixels as wholly infracted or not (2.7 vs 7.7%LVM, P < 0.01) and was not statistically significantly different from inter-observer variability for bias (P = 0.31) or variability (P = 0.38). Also, calculation of weighted infarct transmurality has been implemented and validated [23]. Typical calculation time for automated infarct delineation for a typical data set with 12 slices is about 0.1 s on an ordinary desktop PC.

### I. General image object segmentation tools

Tools for segmentation of general objects have been implemented, and they are based on an approximate fast-level set algorithm [24]. The segmentation can be exported as a three-dimensional mesh. This method has been described in detail already [25]. Moreover, a novel prototype-based segmentation algorithm has been developed. This algorithm is capable of learning how to segment an object based on few examples, typically 5-10. This can be contrasted to traditional statistical model-based approaches, which generally required 50-100 training cases. In a test case the aorta was delineated in 10 healthy volunteers imaged using a steady-state free precession sequence, resolution 1.6 × 1.6 × 1.0 mm. Five volunteers were used as a learning set and five as test case. The volumetric error was 4 ± 5% when measured as volumetric error and 0.58 ± 0.06 mm when measured as distance error.

### J. Left ventricle segmentation in SPECT images

Automated delineation of the left ventricle in myocardial perfusion SPECT has been implemented. The result of the fully automated segmentation in myocardial perfusion SPECT images of 100 patients was compared to that from manual planimetry of MRI images of the same patients as a reference standard. The mean error and variability compared to MRI was 6 ± 15%LVM, which was significantly lower than for the commercially available algorithm Quantitative Perfusion SPECT (QPS, Cedars-Sinai Health Systems): 18 ± 19%LVM [26]. Typical time for fully automated LV segmentation is about 8 s on an ordinary desktop PC.

### K. Image fusion

A tool for manual rigid body co-registration of image stacks was implemented and was evaluated in an imaging study where *ex vivo *data from 19 pigs using myocardial perfusion SPECT and MRI were successfully co-registered [27].

### L. Reformatting image tools

General image tools such as multi-planar reconstruction, re-sampling and rotation have been implemented. Accuracy of re-sampling was validated by re-sampling of computer phantoms with known geometric properties. The image quality of re-sampled images is maximized by using bi-cubic interpolation with anti-aliasing filters. Testing of the general image tools is included in the test script.

### M. Polar plots

Quantitative results of regional functional parameters such as wall thickening and infarct transmurality from viability analysis can be displayed as polar plots, including segmentalisation of the left ventricle according to the 17-segment model endorsed by the American Heart Association [28]. Development and validation of this functionality has been described [29], and an example of how this can be used to register quantitative data in consecutive MRI studies has been published [2].

### N. Export capabilities

A wide range of exportation algorithms were implemented. Export direct to the system clipboard can be done from most graphical user interface panels. Data in the clipboard can then be pasted directly into statistical spread-sheet software such as Microsoft Excel. Tools for batch exportation from multiple files are also implemented. This avoids tedious manual interaction, and it also eliminates manual mistakes in the exportation process.

### O. Communication module

A module to facilitate communication between research groups and to facilitate multi-national and multi-centre studies is included in the software. Since the software is freely available to researchers, all participating sites can use the same software. Images are loaded into the software by the imaging site. Using the software, all images from one CMR examination are (optionally) analysed, anonymised, compressed and electronically transferred to a central server set up by the coordinating centre.

The coordinating centre can control the total image flow by distributing "site keys" to each participating site. The site key includes instructions to the imaging centre and tells the software where images from a specific site should be stored. No passwords or usernames need to be sent out to participating sites. The site key also contains the encryption/decryption key.

### P. Plug-in and scripting capability

Possibilities for writing one's own dedicated plug-ins have been implemented and details are given in the technical manual. A detailed description is outside the scope of this article. A plug-in template is available, consisting of less than 30 lines of Matlab code, and user plug-ins are available directly main menu in Segment. Users have access to the complete internal data structure and have control over all elements of the graphical user interface. Scripting can be performed directly in Matlab, thus providing powerful scripting possibilities.

### Q. Test script

Testing includes user interface, display, and low-level algorithms such as automated LV segmentation, vessel delineation, flow quantification, DE-MRI viability analysis etc. Currently, the test script comprises 66 main test cases and a total of 316 tests. The test script is continuously refined as new features are added to the program. A script that checks for broken call-back links in the user interface is also implemented.

## Discussion

Previous work on extendable software for medical imaging can be divided into two major categories: (1) complete applications, and (2) toolkits such as MITK http://www.mitk.org[1]. These two different approaches to extendable medical analysis software have their own strengths and weaknesses. Early on, we considered whether Segment should be a toolkit or a complete application. It was decided that an application approach would be most beneficial to clinical research since it will allow the great majority of users to be able to use it without having to write any source code. One pioneering and freely available extendable medical imaging software package is ImageJ http://rsbweb.nih.gov/ij/, written by Rasband at the U.S. National Institutes of Health. ImageJ has become widely popular, probably because of the open platform that allows users to write their own plug-ins, thus rapidly increasing the applicability of the software. Another open source general viewing and image processing application that also has a complete plug-in architecture is OsiriX http://www.osirix-viewer.com/, which runs under Mac OS X. Yet another well-known application is Slicer http://www.slicer.org/[30]. One example of extendable software for medical imaging that is not open source is Analyze (AnalyzeDirect, Lenexa, KS; http://www.analyzedirect.com/). The majority of freely available medical research software approaches have been designed for analysis of the brain, such as SPM http://www.fil.ion.ucl.ac.uk/spm/software/spm2/ and Internet Image Viewer http://james.psych.umn.edu/iiV/[31], and not cardiovascular applications.

In summary, Segment differs compared to previous approaches in that to our knowledge it is the first source code extendable software application dedicated for cardiac image analysis. It is also to our knowledge by far the largest solution for applied medical imaging written in Matlab, a computer language that is widely used for image processing research. The strength of Segment lies in the combination of both a clinically applicable tool and a tool that easily can be further expanded by image processing experts and directly be used for clinical research.

### Predicted use of Segment

We predict that Segment will continue to be used by cardiovascular researchers and in research groups with engineering teams. New functionality required to answer clinical research questions will be implemented and made available to the research community. The scripting functionality will enhance and facilitate larger clinical studies that are required to help cardiovascular imaging, and cardiovascular MRI in particular, to become an outcome-based imaging modality in medicine. Our hope is that Segment will function as a bridge between researchers in the field of image processing and researchers in cardiovascular research, both clinical and pre-clinical.

### Importance of validation

Accurate and careful validation is of crucial importance in cardiovascular research. One feature that makes this project stand out from many other freely available medical image analysis software packages is the careful scientific validation that is performed when developing the new algorithms used in the software. However, one must remember that a chain is no stronger than its weakest link, and this is certainly true of quantitative image analysis. One such example is that flow quantification by MRI is validated in Segment, but it has been shown that each MRI scanner is unique; different pulse sequences have to be individually validated for each scanner since some scanners can introduce large sources of errors [18]. Another example is automated segmentation algorithms, which need to be supervised by trained and experienced observers to achieve the highest accuracy in clinically relevant measures.

## Conclusions

Segment is a cardiovascular image analysis software package that has been used in over 40 peer-reviewed scientific publications, indicating that the software has had an impact on cardiovascular research. Segment is a well-validated and comprehensive package that is freely available in an open source format for research purposes.

## Availability and requirements

The project name is Segment and the project home page is http://segment.heiberg.se. Pre-compiled versions of the software will be made available for Windows and Linux. The Matlab source code version of the program requires Matlab R2008a or later. The software is known to run under Mac OS X, but at the moment this is not supported. Segment is freely available for academic investigational research use, provided that relevant original research publications related to the software are cited. The software is also free for educational purposes. The terms of the licence do not generally include trials paid by pharmaceutical companies. For commercial use, Segment is sold and supported by Medviso AB, Lund, Sweden. Individuals or organisations are not allowed to compile software products derived from Segment that are to be sold commercially or shipped together with other commercial products without the express written permission of Medviso AB.

## Abbreviations

DE-MRI: Delayed Enhanced Magnetic Resonance Imaging; MRI: Magnetic Resonance Imaging; LV: Left Ventricle; LVM: Left Ventricle Mass; PACS: Picture Archiving Communication System; SPECT: Single Photon Emission Computed Tomography; CT: Computed Tomography; PET: Positron Emission Tomography.

## Competing interests

The authors declare that they have no competing interests, with the following two exceptions. Einar Heiberg Ph.D. developed most of the Segment software described in this study. Segment is freely available for research use, and it is sold for commercial use by Medviso AB, Lund, Sweden, a company of which Dr. Heiberg is the founder and the major shareholder. Jane Sjögren is employed by Medviso AB on a part-time basis.

## Authors' contributions

EH designed and programmed most of the software and wrote major parts of the paper. JS programmed large parts of the general object segmentation module, designed and implemented the test script, and revised the manuscript for important intellectual content. MU, MC, HE and HA conceived many of the functions in the software and revised the manuscript for important intellectual content. All authors read and approved the final manuscript.

## Pre-publication history

The pre-publication history for this paper can be accessed here:

http://www.biomedcentral.com/1471-2342/10/1/prepub
